# Hyperbaric oxygenation and glucose/amino acids substitution in human severe placental insufficiency

**DOI:** 10.14814/phy2.13589

**Published:** 2018-03-13

**Authors:** Michael Tchirikov, Erich Saling, Gauri Bapayeva, Michael Bucher, Oliver Thews, Gregor Seliger

**Affiliations:** ^1^ Center of Fetal Surgery University Clinic of Obstetrics and Fetal Medicine University Medical Center Halle (Saale) Martin Luther University Halle‐Wittenberg Halle Germany; ^2^ Saling Institute of Perinatal Medicine Berlin Germany; ^3^ National Research Center for Mother and Child Health Nazarbayev University Astana Republic of Kazakhstan; ^4^ Center of HBO University Clinic of Anesthesiology University Medical Center Halle (Saale) Martin Luther University Halle‐Wittenberg Halle Germany; ^5^ Institute of Physiology Martin Luther University Halle‐Wittenberg Halle Germany

**Keywords:** Amino acids, cordocentesis, fetal growth restriction, HBO, hyperbaric oxygenation, intrauterine treatment, intravenous infusion, IUGR, placental insufficiency, port implantation

## Abstract

In the first case, the AA and glucose were infused through a perinatal port system into the umbilical vein at 30 weeks' gestation due to severe IUGR. The patient received daily hyperbaric oxygenation (HBO, 100% O_2_) with 1.4 atmospheres absolute for 50 min for 7 days. At 31^+4^ weeks' gestation, the patient gave birth spontaneously to a newborn weighing 1378 g, pH 7.33, APGAR score 4/6/intubation. In follow‐up examinations at 5 years of age, the boy was doing well without any neurological disturbance or developmental delay. In the second case, the patient presented at 25/^5 ^weeks' gestation suffering from severe IUGR received HBO and maternal AA infusions. The cardiotocography was monitored continuously during HBO treatment. The short‐time variations improved during HBO from 2.9 to 9 msec. The patient developed pathologic CTG and uterine contractions 1 day later and gave birth to a hypotrophic newborn weighing 420 g. After initial adequate stabilization, the extremely preterm newborn unfortunately died 6 days later. Fetal nutrition combined with HBO is technically possible and may allow the prolongation of the pregnancy. Fetal‐specific amino‐acid composition would facilitate the treatment options of IUGR fetuses and extremely preterm newborn.

## Introduction

Severe intrauterine growth restriction (IUGR), caused by placental insufficiency, is a serious prenatal condition (Haram et al. 2006; Nardozza et al. 2017; Tang et al. 2017). It is often associated with arterial and venous blood flow redistribution which maintains the delivery of oxygenated blood to the brain, or the “brain sparing effect”, and the reduction in the placental arterial blood supply to the fetal liver due to increased shunting through the ductus venosus (Arbeille et al. 1987; Tchirikov et al. 1998, 2006; Cahill et al. 2014). However, a reduced liver blood supply could worsen fetal growth (Tchirikov et al. 2001, 2002, 2006). This reduction in blood flow resistance in the cerebral arteries is associated with significantly increased risk of intraventricular hemorrhage, periventricular leukomalacia, hypoxic ischemic encephalopathy, necrotizing enterocolitis, bronchopulmonary dysplasia, sepsis and death (Flood et al. 2014). Placental insufficiency is responsible for about 40% of all stillbirths (Mongelli and Gardosi 2000; Platt 2014). The long‐term neurocognitive deficits of IUGR include poor executive functioning, cognitive inflexibility with poor creativity and language problems (Figueras et al. 2011).

Treatment options for IUGR are limited and usually necessitate an early delivery (Haram et al. 2006; Mandruzzato et al. 2008; Thorne et al. 2014). There is evidence that the active placental transport of amino acids, glucose, and oxygen from the mother to the fetus is reduced in IUGR fetuses (Cetin et al. 1990, 1992; Rizzo et al. 1995; Jansson et al. 2002; Benirschke et al. 2013). Fetal amino acid and glucose supplementation combined with hyperbaric oxygenation (HBO) could lead to improved fetal growth, and prolonged pregnancy (Xiao et al. 2006; Shyu et al. 2008; Wu 2009; Tchirikov et al. 2010a, 2017).

Previously, we observed inadequate improvement in IUGR fetuses at a gestational age below 28 weeks’ gestation, using intraumbilical amino acid/glucose supplementations via a port system (Tchirikov et al. 2010b). We hypothesized that the additional nutrient load of IUGR fetuses could lead to lactic acidosis, which subsequently led us to use of HBO. HBO can increase oxygen diffusion in the placenta improving energy metabolism in the fetus. In this report, we describe in detail the treatment options of severe IUGR human fetuses with pronounced placental insufficiency and brain sparing, using the combination of HBO with the long‐term administration of nutrients directly into the umbilical vein via a subcutaneously implanted intraumbilical perinatal port system, or transplacental fetal supply.

## Case 1

A 22‐year‐old second gravida, first para patient was referred to the clinic at 30 weeks’ gestation because of severe IUGR and preeclampsia. Three years earlier, the patient had a preterm delivery by C‐section because of severe preeclampsia and brain sparing. The patient did not have any other relevant history (e.g., smoking, etc.). On admission, the patient did not have any complaints. Patient monitoring included the evaluation of a multi‐vessel Doppler examination of the pulsatility index (PI) in the umbilical artery (UA), middle cerebral artery (MCA), uterine arteries (Ut.A), ductus venosus (DV), and CTG. All data were compared with published, standardized references for various Doppler parameters used in ultrasound software (Viewpoint, GE).

The estimated fetal weight was 1022 g (<3rd percentile), with a reduced amount of amniotic fluid (AFI 8.9 cm) (Voluson E8 Expert, GE, Milwaukee, WI). The Dopper parameters reflected placental insufficiency and brain sparing (Table 1). The patient received antihypertensive therapy with methyldopa 250 mg given orally, 4 times daily, and a prophylaxis for the respiratory distress syndrome (RDS) of dexamethasone. It was possible to stabilize the arterial pressure below 140/90 mmHg. The patient was informed about the study on amino acid supplementation via a subcutaneously implanted port system combined with hyperbaric oxygenation. The protocol for the port implantation was approved by the institutional review board and the invasive procedures were performed with written informed consent by the patient.

**Table 1:** Ultrasound, Doppler and CTG examinations before and after HBO treatment

Parameter	Case 1 before HBO 30 WG	Case 1 after 5 HBOs 31 WG	Case 1 after 7 HBOs 31^+2^ WG	Case 2 before HBO 25 WG	Case 2 immediately after HBO
Fetal weight	1022 g	–	1378 g	470 g	–
Ut. A.‐PI	0.79/1.97	0.81/2.04	0.68/1.64	2.9/3.3	1.9/2.05
UA‐PI	2.99	1.4	3.5	3.27	3.0
MCA‐PI	0.99	1	1.14	1.12	0.93
DV‐PI	0.55	–	–	0.4	1.0
CTG‐STV	10.2 msec	9.8 msec	–	3.9 msec	9 msec

PI, pulsatility index; UA, umbilical artery; MCA, middle cerebral artery; Ut.A, the uterine arteries; DV, ductus venosus; CTG, cardiotocography; STV, short time variation; WG, weeks’ gestation.

**Figure 1:**
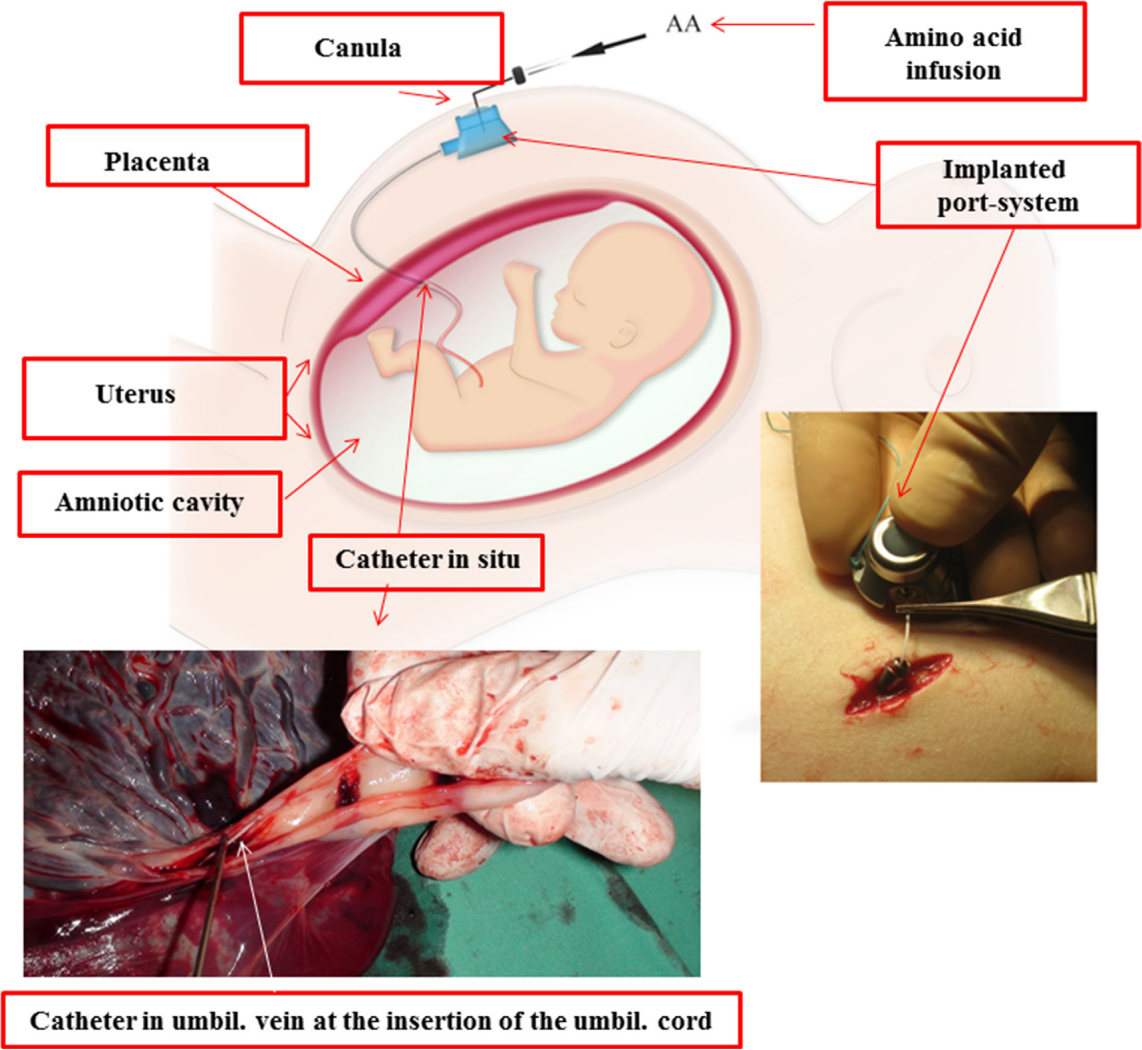
Amino acid and glucose supplementation via a subcutaneously implanted intraumbilical port system. (A) Connection of the port catheter to the port chamber in preparation for transplantation. (B) The situation after port implantation. The 25‐gauge port needle was used to enter the port system. The port system is connected to the pump containing amino acid and glucose solutions (with permission of J Perinatal Med (Tchirikov et al. 2017).

At 30^+1^ weeks’ gestation, the port implantation was performed under local anesthesia without any complications (Tchirikov et al. 2017). For the ultrasound‐guided puncture of the umbilical vein, we used an 18‐gauge needle (Echotip^®^ Disposable Trocar Needle, COOK Medical, Spencer, IN) and the catheter was inserted transplacentally into the umbilical vein and then connected to the port capsule (Fig. 1) (Tchirikov et al. 2017).

The 25‐gauge port needle was changed every 3 days. The pH in the umbilical vein was 7.43. Amino acids (4 mL/day, Aminoven infant 10%, Fresenius Kabi GmbH, Bad Homburg, Germany) and 10% glucose (20 mL/day) were infused with a constant rate of 1.0 mL/h for 9 days. The patient received daily HBO (100% O_2_) with 1.4 atmospheres absolute (ATA) for 50 min for 7 days (Baromed, Perry Baromedical Corporation, Florida). One week later, the resistance to the blood flow in the UA was measured to be slightly improved (Table 1). At 31^+4^ weeks’ gestation, the patient gave birth spontaneously to a preterm newborn weighing 1378 g, with a length 34 cm, pH 7.33, APGAR score 4/6/intubation. The port system was removed under local anesthesia. The pathological examination reported a small, insufficient placenta with infarcts and calcifications. The position of the catheter was found to be optimal without any local hemorrhage (Fig.**** 1). Four days postdelivery, the patient was discharged from the hospital in a healthy condition without any further treatment, and the baby was discharged 3 weeks later. In follow‐up examinations at 5 years of age, the boy was doing well, but the speech development was delayed without any other neurological disturbance.

## Case 2

**Figure 2:**
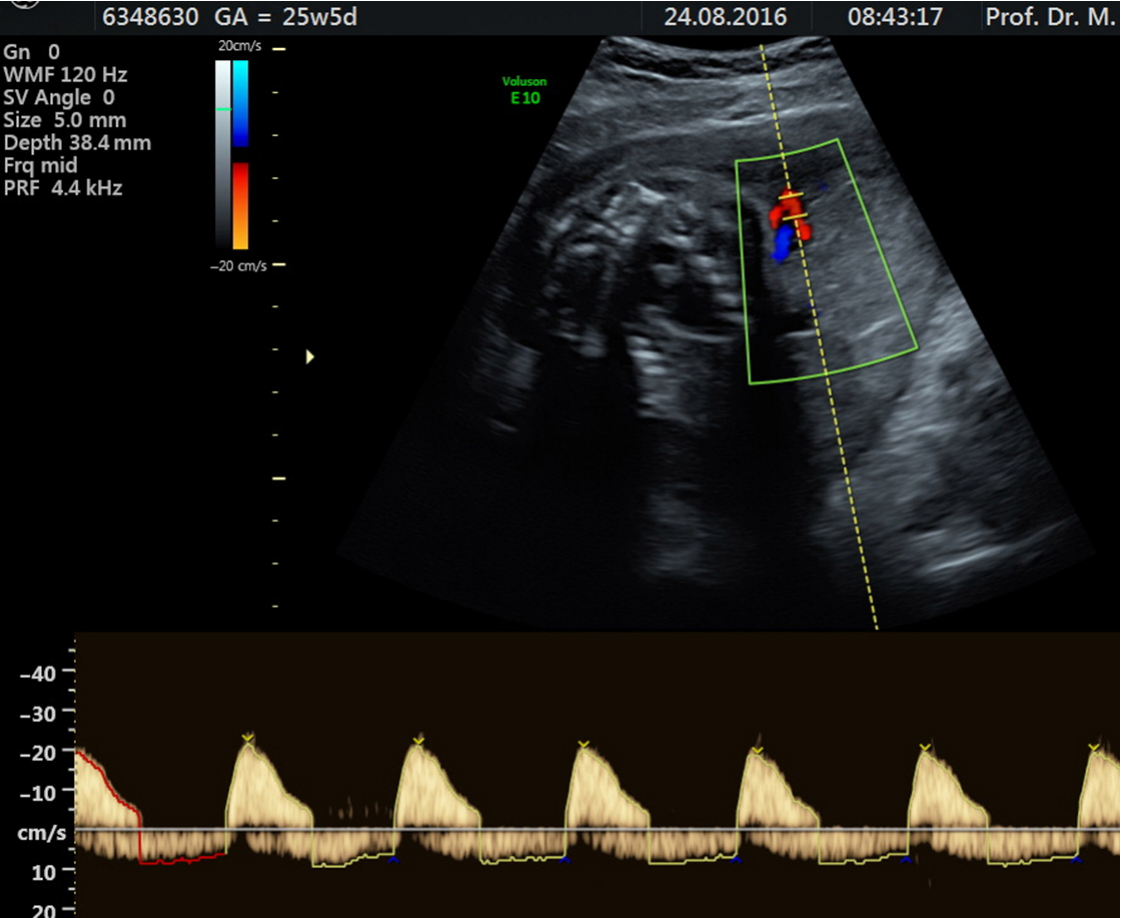
Doppler profiles of the umbilical artery and vein. Note the pulsation of the UV.

A 30‐year‐old third gravida, null para patient was referred to our clinic at 25^/0^ weeks’ gestation because of severe IUGR with brain sparing and preeclampsia. The patient had indicated two previous miscarriages at 9 and 10 weeks’ gestation. The patient was diagnosed 2 years’ previously with systemic lupus erythematosus (SLE), which had been treated with hydroxychlorochine 200 mg/day and prednisolone 5 mg/day. On admission, the fetal weight was estimated to be 470 g (<3rd percentile), and clinical observation included anhydramnios, zero diastolic blood flow in the UA, with strong pulsation of UV (Fig. 2), brain sparing and the notching of both Ut.A and normal DV blood flow profile (Table 1, Viewpoint E10 Expert, GE).

The TORCH test was negative. The monitored RR rate was stabilized below 160/100 mmHg. The patient had a proteinuria >3 g/day. The patient received RDS prophylaxis with 12 mg i.v. betamethasone (two applications over 24 h), 2 L infusion therapy, Aminovent Infant 10% 40 mL/h i.v. with 10% glucose, O_2_ 3 L/min therapy, and an s.c. thrombosis prophylaxis.

The patient was informed about the experimental treatment and the possible potential for HBO and amino acid supplementation. However, the direct intraumbilical administration of AA, using the perinatal port system was not offered because an amino acid solution corresponding to fetal AA concentrations was not available (Tchirikov et al. 2017). Our previous prospective study indicated the intraumbilical delivery of a commercial AA formula known to deviate from fetal AA proportions is not effective in severe IUGR fetuses with brain sparing below 28 weeks’ gestation (Tchirikov et al. 2017). We obtained the informed written consent of the patient and approval from the University Ethics Committee for HBO‐only treatment.

**Figure 3:**
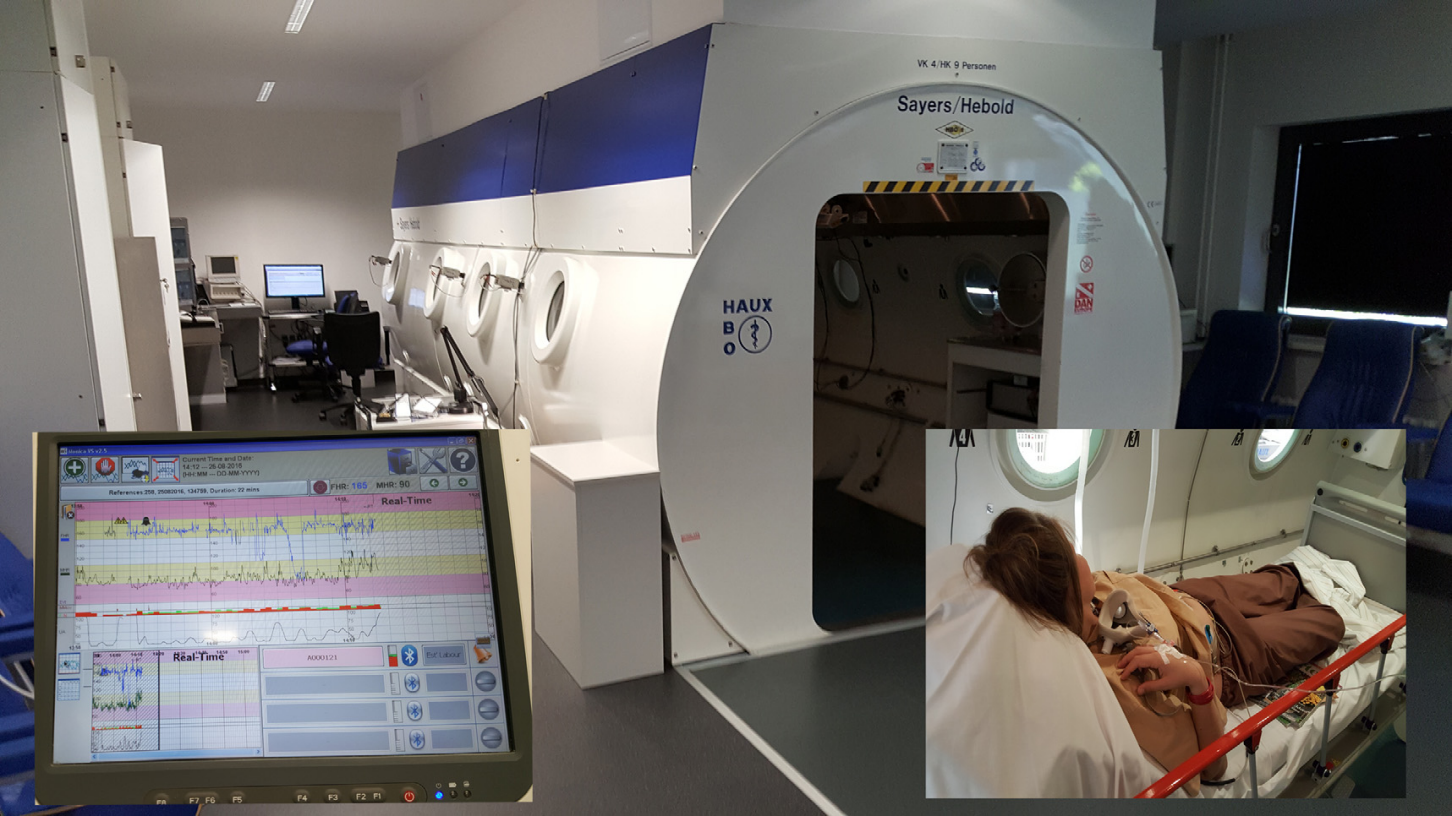
The HBO process. The patient received HBO (Sayers/Hebold, Cuxhaven, Germany) with 1.4 atmospheres absolute for 50 min, maximum transcutaneous oxygen – tcpO_2_ was measured 723 mmHg. Fetal monitoring was performed using a Monica ECG tool affixed to the HBO chamber.

The Ethics Committee stipulated the continuous monitoring of the mother and fetus during HBO. While the mother could be sufficiently monitored inside the HBO chamber, the standard equipment had no capabilities for fetal monitoring. For this reason, our engineer modified the HBO chamber by affixing an ECG‐cable (Monica^®^), which enabled sufficient fetal heart rate and ECG monitoring throughout the entire HBO treatment. The patient received HBO (Sayers/Hebold, Cuxhaven, Germany) with 1.4 atmospheres absolute for 50 min, the maximum transcutaneous oxygen – tcpO_2_ was measured as 723 mmHg (Fig 3).

During HBO treatment, the patient complained of uterine contractions, which could be treated with i.v. tocolysis, using 25 *μ*g fenoterol hydrobromide (Partusisten^®^, Boehringer Ingelheim Pharma KG, Ingelheim, Germany). The Doppler parameter did not change in response to HBO (before/after HBO (Table 1), although the cCTG improved sufficiently, increasing the STV from <2.9 to 9 msec (Fig. 3). One day later, the patient was delivered by cesarean section because of fetal late decelerations. The newborn weighed 420 g, APGAR 3/8/8, art. pH 7.12 and BE – 8.5 mmol/L was intubated and transferred to the neonatal ICU for high flow oxygen/NO treatment and surfactant applications. After an initial adequate stabilization, the newborn unfortunately developed a pulmonary bleed on the 4th postnatal day, with subsequent hypotonia, anuria, leucopenia and thrombocytopenia, and died 6 days later. The pathological examination reported a placenta (11 × 8.7 cm area), weighing 86 g, with large necrotic areas, fibrosis, and calcifications.

## Discussion

IUGR is one of the major causes of preterm delivery, which itself is associated with an increase in perinatal mortality and morbidity, in addition to fetal under‐nutrition and chronic hypoxia (Haram et al. 2006; Goldenberg et al. 2008). The WHO defines preterm delivery as a global problem. The survival rate of extreme preterm infants (<28 weeks gestation) is still low, with 40% dying by the age of 5 years (WHO) (March of Dimes, PMNCH 2012). Fetuses with IUGR have a higher rate of mortality [OR 8.3] as well as perinatal complications [OR 31.6] (Beckerath et al. 2013). The long‐term impacts of extreme preterm birth include: impairment of vision, hearing and executive functions, developmental delay, psychiatric and behavioral problems, cardiovascular and pulmonary diseases, and resulting socioeconomic burdens (March of Dimes, PMNCH 2012; Beckerath et al. 2013; Figueras et al. 2011; Howson et al. 2013; Platt 2014).

The etiology of IUGR is multifactorial and may be subdivided into maternal causes such as hypertension, diabetes, severe heart‐, autoimmune‐, renal‐ or other systemic‐diseases, nicotine and drug abuse, nutritional disorders, fetal causes, like chromosomal abnormalities, mosaicism and genetic syndromes, infections and metabolism‐disorders, and causes involving uteroplacental vascular insufficiency (Nardozza et al. 2017). The placenta is an essential organ for the transfer of nutrients and gases from the mother to fetus and for the elimination of products resulting from fetal metabolism. The interaction between the maternal and fetal circulations in the placenta is fundamental for the adequate exchange of nutrients and oxygen. Because of the presence of paternal antigens, the fetus and the fetal part of the placenta represent an allograft to maternal tissues (Nardozza et al. 2017). The inadequate trophoblast invasion of the myometrial portion of the spiral arteries, the reduced synthesis of nitric oxide and endothelial adhesion molecules in extravillous trophoblast lead to an increased vasoconstricting agent activity, a resistance to blood flow and a resultant decreased nutrition and oxygen transport of the intervillous space (Grati et al. 2017; Nardozza et al. 2017; Tang et al. 2017).

During sustained hypoxia, fetal growth is slow, although oxygen consumption may be unaltered when corrected for fetal mass (Carter 2015). The increased anaerobic metabolism of glucose in the placenta could spare oxygen for the fetus but could reduce its supply of substrate and thereby limits fetal growth (Carter 2015). The mechanisms leading to neuronal injury in the IUGR neonatal brain are complex and not well understood. The brain sparing of IUGR fetuses is associated with changes in neurotransmitter profiles (Garcia‐Contreras et al. 2017). Neuroinflammation elevated production of proinflammatory cytokines and tumor necrosis factor‐*α* (Wixey et al. 2017). IUGR has been linked to reductions in overall brain volumes, with regional changes in gray and white matter volumes (Padilla et al. 2011), significantly thinner insular cortical thickness, and a smaller insular cortical volume than controls (Egana‐Ugrinovic et al. 2014).

As a consequence of irreversible placental pathology, treatment options for IUGR with placental insufficiency remain extremely limited (Haram et al. 2006; Benirschke et al. 2013; Thorne et al. 2014). A number of changes in the activity of amino acid transporters have been identified in the IUGR placenta (Pardi et al. 2002). In IUGR pregnancies, the maternal concentration of most amino acids is significantly higher than in normal pregnancies, which determines a significant increase in total nitrogen by approximately 18%. This observation coupled with lower fetal amino acid concentrations in IUGR fetuses, leads to significantly lower fetal‐maternal amino acid concentration differences in IUGR pregnancies (Cetin et al. 1992; Pardi et al. 2002). The active placental transport of amino acids, glucose and oxygen from the mother to the fetus is reduced due to the altered angiogenesis of placental tissue, disturbed syncytio‐ and cytotrophoblast development, proliferation, and trophoblast invasion (Cetin et al. 1992; Jansson et al. 2002; Benirschke et al. 2013). It is impossible for any treatment of IUGR to replace an organ as complex as the human placenta. However, a partial therapeutic solution to IUGR could be the supply of growth‐restricted fetuses with amino acids and glucose under HBO conditions. This could lead to improved fetal growth and prolong pregnancy (Tchirikov et al. 2010a, 2017). Paz et al. (2001) were able to demonstrate that full‐term infants with fetal growth restriction are not at increased risk for low intelligence scores at the age 17.

The intrauterine application of amino acids and glucose into the amniotic fluid for the treatment of IUGR human fetuses was first developed and introduced by Saling and his coworkers in the 1970s (Dudenhausen et al. 1972; Saling 1972, 1987). Intraamniotic amino acid application increased the amino acid concentration in fetal plasma but the increased risk of amnion infection syndrome thwarted the use of this method (Saling 1987).

**Figure 4:**
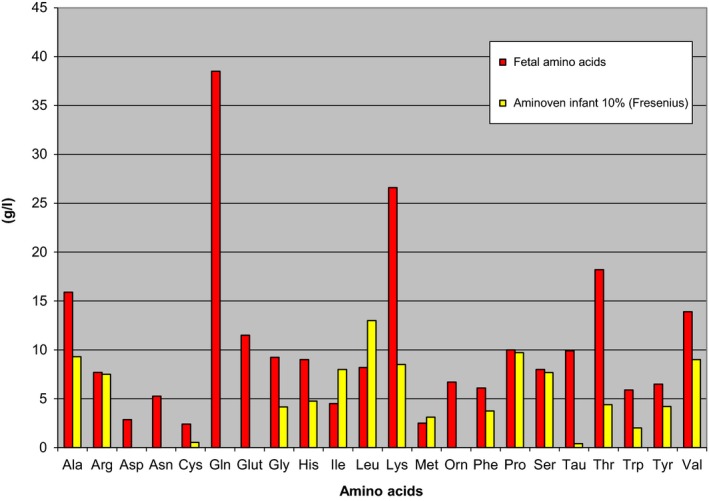
The amino acid concentrations of the commercial amino acid solution vs. amino acid concentrations observed in very preterm human fetuses under physiologic conditions. The commercial amino acid solution used was Aminoven infant 10% (Fresenius Kabi GmbH, Bad Homburg, Germany). The required amino acids’ (AA) concentration related to plasma AA concentration of human fetuses under physiological conditions was calculated as AA =  fetal AA concentrations (mean) × 500 (Economides et al. 1989; Cetin et al. 1990, 1992; Tchirikov et al. 2017). The commercial AA solution did not contain aspartic acid, glutamic acid and ornithine. Furthermore, this solution had fourfold lower relative concentrations of lysine, and threonine, and 25‐fold lower relative concentration of taurine, compared to the physiologic AA proportions in the plasma of extreme preterm fetuses.

We used the perinatal port system for the long‐term administration of nutrients into the umbilical vein of the first patient without any complications. In our previous study, we reported that intraumbilical supplementation, using a commercial amino acid solution via a port system did not lead to a sufficient weight gain in extremely preterm IUGR fetuses (Tchirikov et al. 2017). We hypothesized that the additional nutrient load of IUGR fetuses could lead to lactic acidosis. On the other hand, commercial amino acid solutions significantly deviate from the amino acid proportions in normal fetal plasma (Fig. 4) (Economides et al. 1989; Cetin et al. 1990, 1992), which could worsen the amino acid imbalance of IUGR fetuses (Fig. 4). The commercial AA solution used did not contain aspartic acid, glutamic acid or ornithine. Furthermore, the solution had fourfold lower relative concentrations of lysine and threonine and 25‐fold lower relative concentration of taurine, compared to the physiologic AA proportions in the plasma of extreme preterm fetuses. For these reasons, we did not use the intraumbilical port system to treat the second patient who presented at 25 weeks’ gestation.

Hyperbaric oxygen (HBO) therapy is defined by the Undersea and Hyperbaric Medical Society (UHMS) as a treatment in which a patient intermittently breathes 100% oxygen under a pressure that is greater than the pressure at sea level [a pressure greater than 1 atmosphere absolute (ATA)]. HBO has been shown to be a potent means of increasing the oxygen content of blood and has been advocated for the treatment of various ailments, including air embolism, carbon monoxide poisoning, wound healing, and ischemic stroke affecting the expression of the endothelial adhesion molecules, NO production, NOS expression, cellular energetics, lipid peroxidation, and microvascular blood flow. (Buras 2000; Calvert et al. 2007). HBO with a significantly higher pressure is used as a standard procedure during pregnancy as a therapeutic option in patients with carbon monoxide intoxication (Kao and Nañagas 2004). Wattel et al. (2013) did not find any deviations in psychomotor or height/weight criteria of children following HBO treatment for carbon monoxide (CO) poisoning during pregnancy.

We used HBO with only 1.4 atmospheres absolute for 50 min (real time of 75 min with HBO initiation and completion). Modern HBO treatment is a relatively safe method. Analysis of 1.5 million HBO treatments by Jokinen‐Gordon et al. (2017), found that only 0.68% of treatments were associated with an adverse event. Barotrauma and confinement anxiety were the most frequently reported events. Similarly, Hadanny et al. (2016) reported a complication rate of 0.72% when analyzing the adverse effects of HBO on 2334 patients. Long et al. (2017) found that the HBO therapy is safe and effective for the treatment of sleep disorders in children with cerebral palsy.

The second patient reacted with increased uterine contractions to the HBO. The possible constricting influence of HBO onto arterial vessels is common (Thews and Vaupel 2016; van der Bel et al. 2017). We have not registered any increased uterine contraction of pregnant patients with carbon monoxide intoxication treated with HBO in our medical center till now. van Hoesen et al. (1989) described also a good tolerance of HBO in pregnant patients.

The influence of HBO on tissues and metabolic and regeneration processes remains controversial. Oxidative stress, an imbalance between free radical generation and antioxidant defense, is recognized as a key factor in the pathogenesis of adverse pregnancy outcomes (Sultana et al. 2017). Exposure to HBO could increase the formation of oxygen radical species and reduce antioxidant enzyme activity, causing lipid peroxidation and DNA damage (Chavko and Harabin 1996; Gröger et al. 2009; Michalski et al. 2011; Simsek et al. 2011; Yuan et al. 2011; Schmale et al. 2012; Wixey et al. 2017). Yuan et al. (2011) demonstrated limited DNA damage in response to HBO with 2.2 ATA in human umbilical cord endothelial cells. In opposition to this finding, Migita et al. (2016) demonstrated that HBO (>2 atmospheres absolute) suppressed apoptosis, which caused inflammation after renal ischemia/reperfusion, and promoted tubular cell regeneration. Zeng et al. (2012) found that HBO preconditioning could significantly increase the level of the cortical neurons’ peroxisome proliferator‐activated receptor‐*γ* mRNA, playing a central role in the regulation of apoptosis and oxidative stress.

Huang et al. (2016) who applied HBO 1 h/day × 3 days at 2 ATA were able to demonstrate in a rat model that HBO significantly decreased MRI‐identified abnormalities and tissue histopathology after repetitive mild traumatic brain injury. Yu et al. (2015) demonstrated that early HBO (2 ATA for 1 h) after CMA occlusion could have protective effects on brain tissue after cerebral ischemia, possibly via the inhibition of tumor necrosis factor‐alpha and phospho‐protein kinase C‐alpha. Wei et al. (2015) suggested that HBO promotes neural stem cell proliferation and protects learning and memory abilities in neonatal hypoxic‐ischemic brain damage. Chen et al. (2016) demonstrated that HBO enhances antioxidant capacity and reduces the ultrastructural damage induced by hypoxic‐ischemia, which may improve synaptic reconstruction and alleviate immature brain damage to promote the habilitation of brain function.

Shyu et al. (2008) showed that HBO can induce the expression of a placental growth factor in human bone marrow‐derived mesenchymal stem cells, which may play an important role in HBO‐induced vasculogenesis. The combination of HBO with glutamine substitution was effective in reducing neuronal apoptosis, increasing serum prealbumin concentration and improving neurological function following traumatic brain injury (Fu et al. 2014). The use of HBO has been recently described in artificial uterus system and the placenta supporting oxygen supply of very preterm neonate in parallel to ECMO equipment (Tchirikov 2017).

In conclusion, the combination of HBO and intraumbilical amino acid and glucose supplementations via a port system or transplacental supply, offer a treatment option in the development of placenta substitution in cases with severe IUGR. We believe that the reestablishment of fetal physiological concentrations of amino acids, glucose, trace elements, hormones, growth factors, vitamins, and oxygen in IUGR fetuses, using HBO and a placental by‐pass of these substances via an intraumbilical port system could improve neonatal outcomes and IUGR‐altered fetal programing.

## Conflict of Interest

The authors do not have any conflicts of interest or financial disclosure.
